# Glycyrrhetinic Acid Protects α-Naphthylisothiocyanate- Induced Cholestasis Through Regulating Transporters, Inflammation and Apoptosis

**DOI:** 10.3389/fphar.2021.701240

**Published:** 2021-09-24

**Authors:** Miao Yan, Lin Guo, Yan Yang, Bikui Zhang, Zhenyan Hou, Yue Gao, Hongmei Gu, Hui Gong

**Affiliations:** ^1^ Department of Pharmacy, The Second Xiangya Hospital, Central South University, Changsha, China; ^2^ Department of Pharmacy, The Affiliated Yantai Yuhuangding Hospital of Qingdao University, Yantai, China; ^3^ Department of Pharmaceutical Sciences, Beijing Institute of Radiation Medicine, Beijing, China; ^4^ Chia Tai Tianqing Pharmaceutical Group Co. Ltd., Lianyungang, China

**Keywords:** glycyrrhetinic acid, cholestasis, farnesoid x receptor, apoptosis, Nrf2/Keap1, inflammation

## Abstract

Glycyrrhetinic acid (GA), the active metabolic product of Glycyrrhizin (GL) that is the main ingredient of licorice, was reported to protect against α-naphthylisothiocyanate (ANIT)- induced cholestasis. However, its protective mechanism remains unclear. In our work, the cholestatic liver injury in mice was caused by ANIT and GA was used for the treatment. We assessed cholestatic liver injury specific indexes, histopathological changes, bile acid transporters, inflammation and apoptosis. The results of liver biochemical index and histopathological examination showed that GA markedly attenuated ANIT-induced liver injury. Mechanism research suggested that GA could activate the expression of farnesoid x receptor (FXR) and its downstream bile acids transporters Na^+^/taurocholate co-transporting polypeptide (NTCP), bile salt export pump (BSEP) and multidrug resistance-associated protein 2 (MRP2), as well as the nuclear factor erythroid 2-related factor 2 (Nrf2) and its downstream proteins MRP3, MRP4. These transporters play a vital role in mediating bile acid homeostasis in hepatocytes. Moreover, GA could significantly inhibit the ANIT-induced activation of the nuclear factor kappa-light-chain-enhancer of activated B cells (NF-κB) inflammatory pathway and the increase of tumor necrosis factor-α (TNF-α) concentration in serum. Also, GA protected against ANIT-induced mitochondrial apoptosis by regulating the expression of Bcl-2, Bax, cleaved caspase 3 and cleaved caspase 9. In conclusion, GA alleviates the hepatotoxicity caused by ANIT by regulating bile acids transporters, inflammation and apoptosis, which suggests that GA may be a potential therapeutic agent for cholestasis.

## Introduction

Cholestasis is a clinical syndrome characterized by impaired bile flow and retention of bile acids in the liver and body, resulting from mechanical or functional obstruction of the hepatobiliary system. As the disease progresses, cholestasis will ultimately result in hepatic fibrosis, cirrhosis, or liver failure (Jansen et al., 2017). Thus, early intervention is urgent. In current clinical practice, ursodeoxycholic acid (UDCA), obeticholic acid and ademetionine are recognized as effective medicines. However, their efficacy and safety are not satisfactory (Wagner and Fickert, 2020). Therefore, identifying new therapeutic approaches is critical.

Licorice, a widely used herbal medicine in traditional Chinese medicine, is known for its hepatoprotective and detoxifying properties (Li et al., 2019). Glycyrrhizin (GL) is the main ingredient of licorice. Glycyrrhetinic acid (GA) is the active metabolic product of GL. It has been reported that GA has a powerful protective effect in multiple liver injuries, like hepatitis (Shi et al., 2020), acetaminophen-induced acute liver damage (Yang et al., 2017) and cholestasis (Zhai et al., 2007; Wu et al., 2018). However, its protective mechanism on cholestatic liver injury remains unclear.

The accumulation of hydrophobic bile acid has been recognized as the predominant mechanism of cholestatic liver injury (Yang et al., 2019). The toxic bile acids may cause dysregulation of bile acid transporters (T. Li et al., 2020a), inflammation (Li et al., 2017), and apoptosis (Yao et al., 2017) in hepatocytes. Therefore, our study has focused on the aforementioned cellular events to confirm the protective effects of GA on ANIT-induced cholestasis and to explore the possible mechanisms.

## Materials and Methods

### Antibodies and Chemical Reagents

GA (purity≥97%) and ANIT (purity≥98.5%) were purchased from Xiya Reagent Co., Ltd. (Shandong, China). Corn oil was purchased from Solarbio Technology Co., Ltd. (Beijing, China). Antibodies to nuclear factor erythroid 2-related factor 2 (Nrf2, AB89443), Bcl-2 (AB692), Bax (AB32503) and cleaved caspase 3 (AB2302) were purchased from Abcom (MA, USA). Antibodies to farnesoid x receptor (FXR, SC25309) and multidrug resistance-associated protein 2 (MRP2, SC59611) were purchased from Santa Cruz Biotechnology (CA, United States). Antibodies to the phosphorylated inhibitor of kappa-B kinase alpha/beta (p-IKKα/β, AF3013), the phosphorylated inhibitor of kappa-B alpha (p-IκBα, AF2002), cleaved caspase9 (AF5240) and bile salt export pump (BSEP, DF9278) were purchased from Affinity Biosciences (OH, United States). Antibodies to Kelch-like ECH-associated protein 1 (Keap1, A17062), nuclear factor kappa-light-chain-enhancer of activated B cells (NF-κB) p65 (A19653), sirtuin 1 (Sirt1, A11267), multidrug resistance-associated protein 3 (MRP3, A9849), multidrug resistance-associated protein 4 (MRP4, A2198), Na^+^/taurocholate co-transporting polypeptide (NTCP,A12721), β-actin (AC026) and Histone H3 (A2348) were purchased from Abclonal Technology (Hubei, China).

### Animal and Experimental Design

Healthy male ICR mice (18–20 g) were purchased from Hunan Slack Jingda Experimental Animal Co., Ltd. (Hunan, China). The mice were domesticated in a standard conditioned environment and had free access to food and water. The animal experiment was approved by the Institutional Animal Care and Use Committee of Central South University (Number:2019sydw0148).

Twenty-four mice were randomly assigned to three groups: control group; ANIT group, only given ANIT (75 mg/kg); GA group, given ANIT (75 mg/kg) and GA (50 mg/kg, 106 umol/kg). The mice received either corn oil or GA *via* intraperitoneal injection once daily for 6 days consecutively. A single dose of ANIT was only given on the 5th day *via* oral gavage in the ANIT group and GA group. On the 7th day, the mice were sacrificed to collect blood and livers.

### Hepatotoxicity Assessments

Blood samples were centrifuged for 10 min (3,000 r/min, 4°C) to obtain serum. The degree of liver injury was assessed by hematoxylin and eosin (HE) staining and serological biomarkers, including alanine aminotransferase (ALT), aspartate aminotransferase (AST), alkaline phosphatase (ALP), total bilirubin (TBIL), direct Bilirubin (DBIL) and total bile acid (TBA) analyzed on an automatic clinical analyzer (7,600, HITACHI Ltd. Tokyo, Japan).

### Measurement of Hepatic Antioxidant Indexes and Malondialdehyde (MDA)

An appropriate amount of liver tissues was grinded with 9 times homogenate medium, then centrifuged for 10 min (3,000 r/min, 4°C) to obtain the supernatant. Subsequently, the supernatant was used to determine glutathione peroxidase (GPx, A005), superoxide dismutase (SOD, A001-1), MDA (A003-1) levels by commercially available kits (Jiancheng Bioengineering Institute, Nanjing, China) according to the manufacturers’ instructions.

### Enzyme-Linked Immunosorbent Assay

Serum tumor necrosis factor-α (TNF-α, 88–7,324–88, invitrogen) and interleukin-1β (IL-1β, 70-EK201BHS-96, Multisciences) contents were analyzed using ELISA kits. Briefly, the serum, standards, and black were prepared and added to the assay plate for 1 h at 37°C. After incubation, biotinylated-specific antibody, enzyme conjugate, chromogenic substrate and stop solutions were added in sequential order. The optical density (OD) at 450 nm was measured by a microplate reader (Epoch, BioTeK, United States).

### Western Blot

Total proteins were homogenized with RIPA buffer (P0013B, Beyotime Biotech, Shanghai, China). Nuclear proteins were prepared with subcellular structure nuclear and cytoplasmic protein extraction kit (AR0106, Boster Biotech, Hubei, China) according to the manufacturer’s instruction.

After denaturation, the same amounts of protein were electrophoresed on 8–12% SDS-PAGE and transferred to PVDF membranes. The membranes were incubated overnight at 4°C with primary antibodies. Subsequently, membranes were washed and incubated with HRP-conjugated secondary antibodies (Boster Biotech, Hubei, China). Chemiluminescence was visualized with an ECL kit (P2200, NCM biotech, Suzhou, China) according to the manufacturer’s protocol.

### Statistical Analysis

All data were analyzed by SPSS 20.0 (SPSS Inc., Chicago, IL, United States) and then graphed as mean ± S.E.M. using GraphPad Prism 6.0 (GraphPad Software Inc. CA, United States). One-way ANOVA with LSD post hoc tests was applied to test for significance between groups (*p*<0.05). ^*^ *p*<0.05, ^**^ *p*<0.01, ^***^ *p*<0.001 vs. control group. ^#^ *p*<0.05, ^##^ *p*<0.01, ^###^ *p*<0.001 vs. ANIT group.

## Results

### Protective Effects of GA Against ANIT-Induced Cholestasis in Mice

**FIGURE 1:**
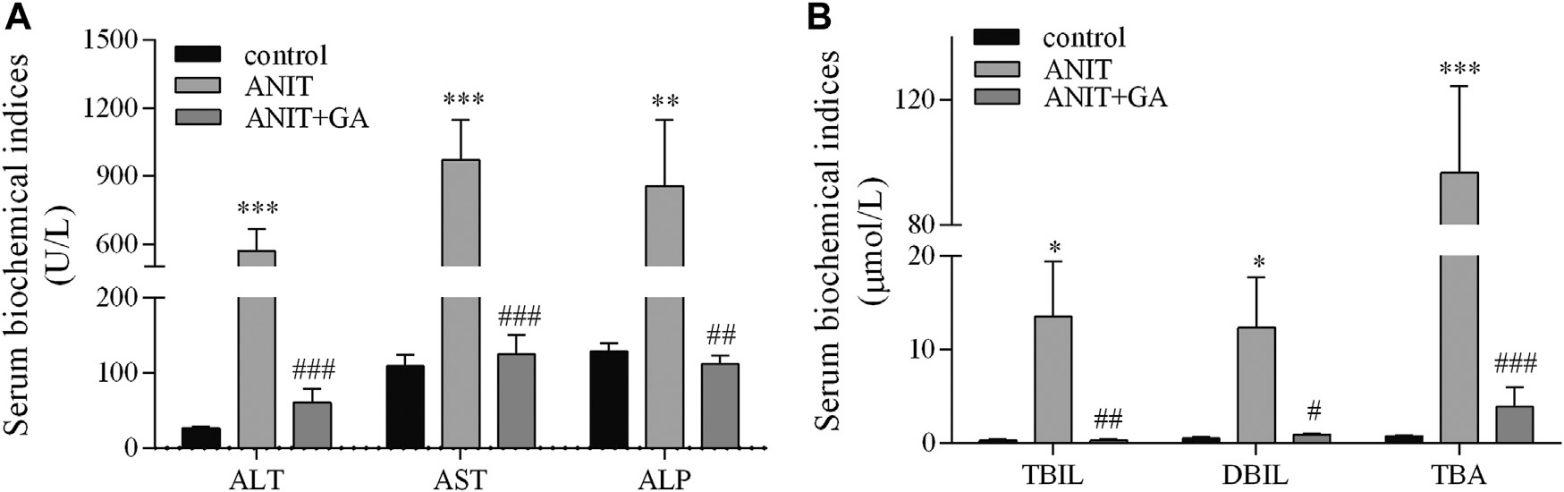
Effects of GA on serological biomarkers of liver injury and biliary cell damage in ANIT-treated mice (**A** and **B**, n=7–8).

To evaluate the protective effects of GA on ANIT-induced cholestatic liver injury, some biochemical indicators were analyzed. As shown in Figure 1, the serum levels of ALT, AST, ALP, TBIL, DBIL and TBA in the ANIT group were increased by 20.5-, 7.9-, 5.6-, 43.9-, 21.7- and 122.1-fold, respectively. When combined GA treatment, all of the above factors were markedly decreased by 89.4, 87.1, 86.9, 97.6, 92.6, and 96.0%, respectively.

**FIGURE 2:**
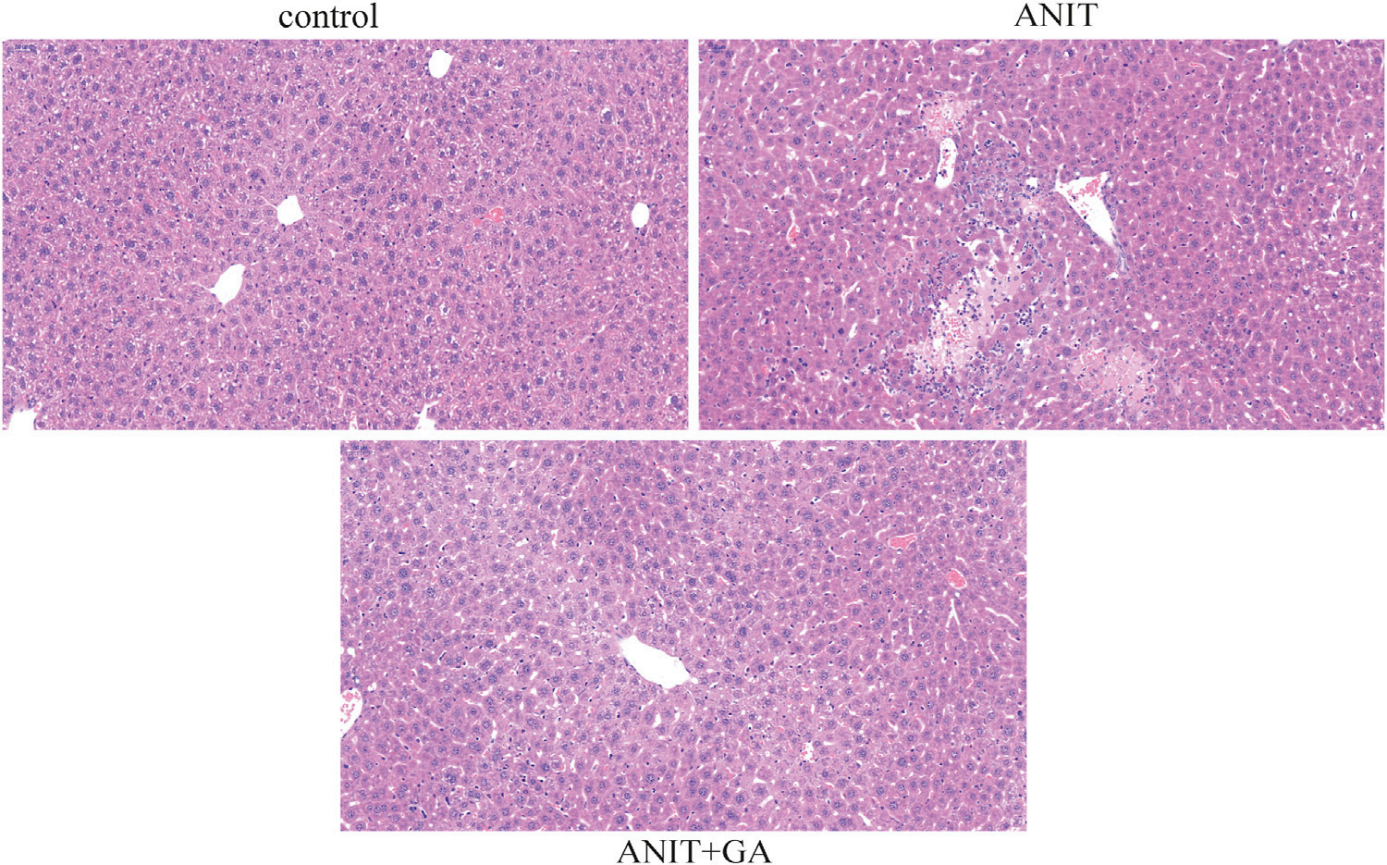
Effects of GA on histological changes in ANIT-induced cholestasis mice (HE stain, ×20).

HE staining of liver sections showed compared with the control group, multiple focal hepatocyte necrosis, nucleolysis and a small amount of neutrophil infiltration in the ANIT group. Whereas the morphological change was significantly ameliorated in the GA group, showing well-arranged hepatic lobules and hepatic cords, no evidence of inflammation, but more hepatocytes with mild cytoplasmic looseness around the central vein **(** Figure 2).

**FIGURE 3:**
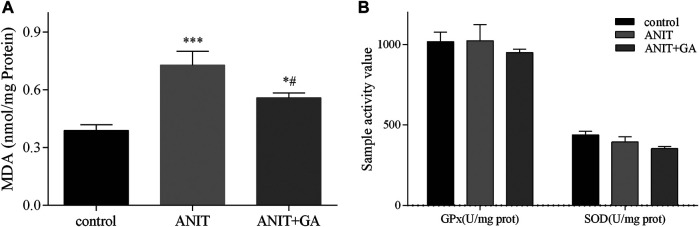
Effects of GA on GPx, SOD **(B)**, and MDA **(A)** in liver specimens in ANIT-induced cholestasis mice (n=7–8).

Also, we measured MDA production, which indirectly reflected the degree of hepatocyte damage. As shown in Figure 3A, the hepatic MDA content in the ANIT group significantly increased, and combined GA treatment could markedly reverse the ANIT-induced increase. But compared with the control group, no difference in GPx and SOD activities was seen after ANIT or combined GA treatment in Figure 3B, indicating that alleviation of ANIT-induced cholestasis by GA was not mediated *via* activation of antioxidant enzymes. These results provided direct evidence that GA could effectively attenuate ANIT-induced cholestatic liver injury.

### GA Altered the Expression of Hepatic Transporters Associated With Bile Acid Homeostasis

**FIGURE 4:**
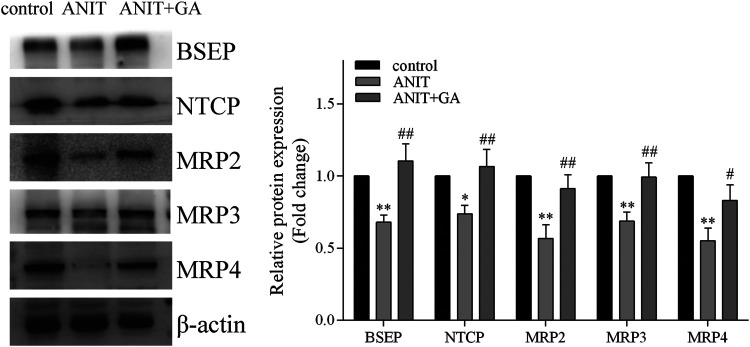
GA alters protein expression of BSEP, NTCP, MRP2, MRP3, and MRP4 in ANIT treatment (*n* = 6).

To elucidate the mechanisms of GA protecting against ANIT-induced liver injury, we measured the protein expressions of transporters associated with bile acid homeostasis. As shown in Figure 4, ANIT could significantly decrease the protein levels of BSEP, NTCP, MRP2, MRP3 and MRP4, whereas protein expressions of all these factors were notably up-regulated to a normal level by GA.

### GA Regulated the Expression of Hepatic Transporters *via* Activating the Sirt1/FXR/Nrf2 Pathway

**FIGURE 5:**
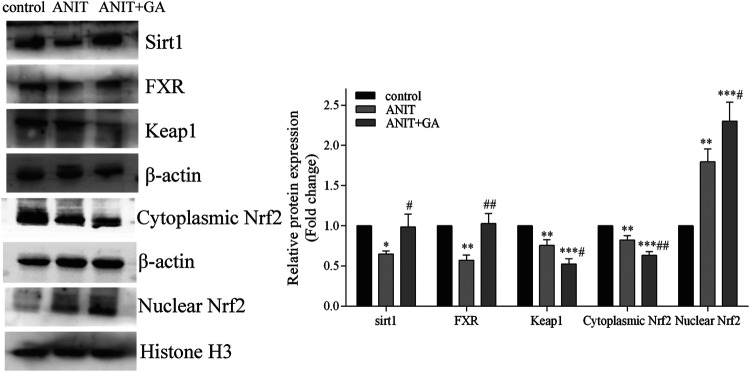
Effects of GA on the expression of Sirt1/FXR/Nrf2 signaling pathway (*n* = 6).

Documents are showing that FXR is a key regulator of NTCP, BSEP and MRP2 (Wei et al., 2021). Nrf2 regulates the expression of MRP3 and MRP4 (Cornejo et al., 2013). Besides, FXR and Nrf2 can be regulated by Sirt1 (Yan et al., 2019). Therefore, we investigated the effect of GA on the expression of these three factors. As exhibited in Figure 5, in comparison to the control group, the protein expressions of FXR and Sirt1 were significantly inhibited in the liver tissue of the ANIT group. GA markedly reversed the ANIT-induced decrease of these two molecules. And western blotting also showed that ANIT significantly inhibited the expression of Keap1 and cytoplasmic Nrf2, and increased nuclear translocation of Nrf2. To our surprise, GA cotreatment further down-regulated Keap1 expression and cytoplasmic Nrf2 level, and up-regulated the nuclear expression of Nrf2 compared with those in the ANIT group (Figure 5).

### GA Inhibited ANIT-Induced Activation of NF-κB Inflammatory Pathway

**FIGURE 6:**
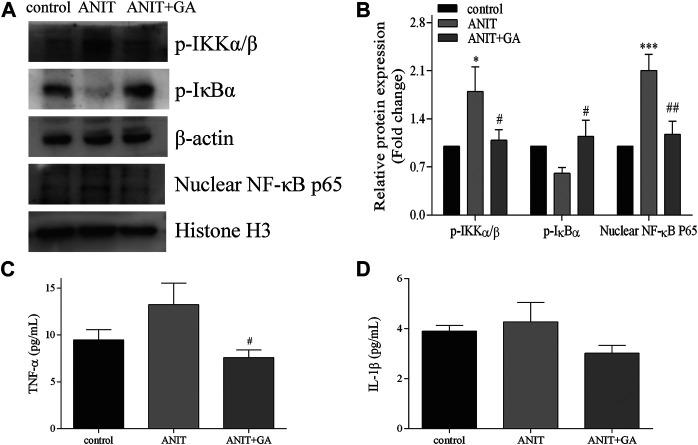
Effects of GA on the expression of NF-κB signaling pathway. **(A)** Effects of GA pretreated on the protein expression of p-IKKα/β, p-IκBα and NF-κB p65 after ANIT treatment. **(B)** The quantitative densitometric analysis of the protein bands (*n* = 6) **(C–D)** Serum contents of TNF-α and IL-1β (*n* = 6–7).

Inflammation is considered to be one of the key causes of cholestasis, so we further mainly measured the activation of the NF-κB inflammatory signaling pathway. As shown in Figures 6A,B, compared with the control group, the phosphorylation level of IKKα/β and the nuclear expression of NF-κB p65 in the ANIT-treated group were significantly higher. GA cotreatment significantly decreased the protein expressions of these two factors. Besides, the expression of p-IκBα in the GA group was significantly higher than that in the ANIT group. We also detected the activities of pro-inflammatory factors regulated by the NF-κB pathway. The results showed that the activity of TNF-α increased in the ANIT group, and GA treatment could notably decrease the TNF-α concentration in blood (Figure 6C). However, Figure 6D showed that ANIT did not cause an increase in IL-1β concentration. Taken together, these results indicated that GA significantly restored ANIT-induced activation of the NF-κB inflammatory pathway.

### GA Protected Against ANIT-Induced Mitochondrial Apoptosis

**FIGURE 7:**
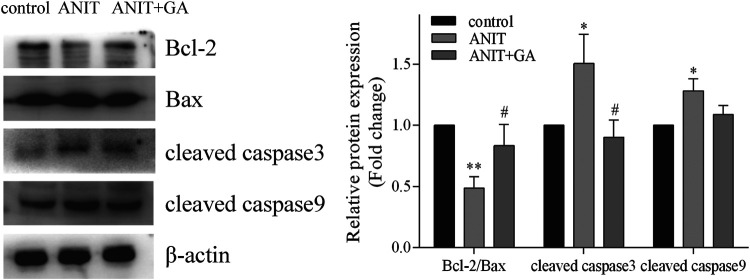
Effects of GA on the expression of the apoptotic signaling pathway (*n* = 6).

To investigate the effects of GA on the mitochondrial apoptotic pathway, we detected the levels of the apoptosis-related proteins such as Bcl-2, Bax, cleaved caspase 3 and cleaved caspase nine by western blot. As shown in Figure 7, we found that ANIT could notably increase the ratio of pro-apoptotic protein Bax and anti-apoptotic protein Bcl-2, the expressions of cleaved caspase 3 and cleaved caspase 9, whereas GA could effectively reverse the ANIT-induced increase in all of the above factors.

## Discussion

The present study demonstrated the effects and mechanism of GA in the prevention of cholestasis induced by ANIT. ANIT is a typical agent which was commonly used for establishing an animal model of intrahepatic cholestasis (Mariotti et al., 2018). ANIT-induced cholestasis is characterized by the elevation of serum biochemical indicators, like ALT, ALP, the necrosis of hepatocytes and biliary epithelial cells and inflammation (Zhao et al., 2017). GA is widely considered as one of the main active substances of licorice. It is demonstrated that GA has protective effects on drug induced liver injury, such as cyclophosphamide (Mahmoud and Al Dera, 2015)and methotrexate (Mahmoud et al., 2017), carbon tetrachloride-induced chronic liver fibrosis (Chen et al., 2013), 2-acetylaminofluorene-induced hepatotoxicity (Hasan et al., 2015), free fatty acid-induced hepatic lipotoxicity (Wu et al., 2008) and ANIT-induced liver damage (Wang et al., 2017). Based on these published research, we chose a single dose of GA which is highly safe and commonly used in mice to confirm its effects on cholestatic liver injury. The outcomes in this study showed that pretreatment of GA remarkably relieved the rising of serum biochemical indicators induced by ANIT, including ALT, AST, ALP, TBIL, DBIL and TBA. Histological examination also demonstrated an obvious improvement in inflammation and necrosis. These results indicated that GA could have a potential therapeutic effect on ANIT-induced cholestasis. However, we should concede that our current work is based on a single dose, which might limit some conclusions drawn from this study, such as effective dose range and correlation between dose and efficacy. Multiple doses will be set up in further investigation.

Based on the premise that this dose of GA is effective, we further focused on the possible mechanism that GA protected against cholestatic liver injury caused by ANIT. Our results demonstrated that the main role of GA on improving cholestatic liver injury was to restore dysregulation of bile acid transporters, inflammation and apoptosis.

Proper regulation of bile acids in hepatocytes is typically important for protecting against cholestatic liver injury (Arab et al., 2017; Trauner et al., 2017). Bile acid transporters play an important role in bile acid homeostasis. NTCP is the major basolateral uptake transporters, functioning to uptake bile acids from plasma. BSEP is responsible for transporting unbound and taurine or glycine salts to the bile duct, while MRP2 is responsible for transporting divalent bile acids such as conjugates with glutathione, sufate and glucuronate (Hua et al., 2021). NTCP, BSEP and MRP2, abundantly expressed at the capillary bile duct membrane of hepatocytes, function as rate-limiting enzymes in bile transportation and excretion (Yang et al., 2018). MRP3 and MRP4 are critical basolateral export transporters, which could promote bile acid exudation into the blood. The increased expression of MRP3 and MRP4 plays a vital role in the protective and adaptive responses to cholestasis. (Perez-Pineda et al., 2021). Pretreatment with GA recovered the expressions of BSEP, MRP2, MRP3, MRP4 remarkably inhibited by ANIT, which may alleviate the accumulation of bile acids. To our surprise, NTCP was also decreased in ANIT group. Maybe it is a compensatory response in our body. It is reported that NTCP may be either increased (Huo et al., 2018) or decreased (Zhao et al., 2017) in cholestasis. It is reported that the expression of NTCP is downregulated in many liver diseases, which may lead to hyperbilirubinemia or exacerbate the current pathological state (Keitel et al., 2005). Our study showed that NTCP expression was reduced by ANIT and subsequently restored by treatment with GA, which could be beneficial to the recovery of bile acid homeostasis.

It is widely believed that FXR serves as a key sensor in the maintenance of bile acids by regulating target genes correlated with the synthesis, detoxification and transportation of bile acids. Bile acid transporters NTCP, MRP2 and BSEP are activated by FXR (Chen et al., 2016). OCA is a potent FXR ligand which was approved for treatment of PBC in patients not responding or intolerant to UDCA (Wagner et al., 2020). Therefore, activating FXR may be a potential therapeutic strategy for cholestatic liver injury. Our results showed that there was a significant down-regulation of FXR in ANIT group, and GA significantly restored the expression of FXR.

Nrf2 is the well-known oxidative stress transcription factor. Under stress conditions, Nrf2 dissociates from its inhibiting factor Keap1 and translocates into the nucleus where it binds to the antioxidant responsive element (ARE), stimulating the expression of target genes. MDA is the end product of lipid peroxidation and its concentration is closely related to the extent of oxidative stress induced cell damage. Generally, excessive ROS in cells can increase production of MDA, resulting in cytotoxicity. It is widely proved that the activation of the Nrf2 signal pathway can induce downstream antioxidant genes expression, such as NAD(*P*)H:quinone oxidoreductase 1 (NQO1), heme oxygenase-1(HO-1) and glutathione-S-transferase (GST), which facilitates to eliminate ROS. As mentioned above, GA reduced the increased production of hepatic MDA, which indicated that GA could moderate the state of oxidative stress through activation of Nrf2. Furthermore, Nrf2 is not only an anti-oxidant defense factor but also the upstream transcription factor of MRP2, MRP3, MRP4 (Cornejo et al., 2013). Emerging evidence has shown that the Nrf2 is important in ANTI-induced cholestasis and liver injury. A previous study showed the improvement of cholestasis induced by lithocholic acid might be due to the upregulation of MRP2, MRP3, MRP4 mediated by Nrf2 (Chen et al., 2015). Therefore, Nrf2 signaling pathway plays a key role in both maintaining systemic bile acid homeostasis and suppressing oxidative stress, and can protect from cholestatic liver injury. Our results showed ANIT induced the nuclear translocation of Nrf2, which may be a Nrf2-independent compensation mechanism (Tanaka et al., 2009). However, GA cotreatment further activated the Nrf2/Keap1 signal pathway through down-regulating Keap1 expression and cytoplasmic Nrf2 level, and up-regulating the nuclear expression of Nrf2.

Sirt1 is an NAD-dependent deacetylase which can regulate multiple cellular processes, including cell proliferation, inflammation, aging and antistress systems (Yan et al., 2019). Deletion of hepatic Sirt1 decreases the HNF-1a/FXR signaling pathways and reduces active biliary excretion of bile acids (Kazgan et al., 2014). In addition, Sirt1 can improve Nrf2 stability by regulating the Nrf2 deacetylation function (Xue et al., 2016). SRT1720, a Sirt1 agonist, has been demonstrated to protect against cholestasis induced by ANIT in mice, which was partly through FXR and Nrf2 activations (Yu et al., 2017). Taken all together, Sirt1 is involved in the activations of FXR and Nrf2, and can be a therapeutic target for the cholestasis treatment. We found the suppression of Sirt1was significantly reversed by the treatment of GA. The above findings indicated that intervention with GA might activate Sirt1, FXR and Nrf2 and their downstream bile acid transporters to relieve cholestasis.

The bile acids of pathologic concentration may cause inflammatory responses (Li et al., 2017). The transcriptional factor NF-κB is recognized as the dominant factor in regulating a series of inflammatory gene expressions. Phosphorylated NF-κB was transported into the nucleus and promoted the release of inflammatory cytokines, such as TNF-α and IL-1β. Studies have shown that a large number of inflammatory cytokines can trigger and amplify local inflammatory response, and eventually, lead to liver injury (Y. Li et al., 2020b). Our results illustrated that GA could alleviate inflammation and liver injury by suppressing the activation of the NF-κB signal pathway and the overproduction of proinflammatory cytokine induced by ANIT.

Apoptosis has been considered as an indirect consequence of bile acids mediated liver injury (Zhou et al., 2017). Inflammatory responses could also aggravate liver damage by inducing apoptosis (Han et al., 2017). Apoptosis, the orderly death of cells, is controlled by a variety of regulatory molecules. Bcl-2 and Bax are involved in the occurrence and progression of apoptosis. Caspase 3 and caspase nine can lead to the rapid induction of apoptosis (Zhou et al., 2017). Our studies showed that GA alleviated ANIT-induced apoptosis by decreasing Bax/Bcl-2 ratio, cleaved caspase3 and cleaved caspase 9.

## Conclusion

In conclusion, our present work confirmed the protective effect of GA on cholestatic liver injury and indicated its possible mechanisms, including regulating the imbalance of transporter and inhibiting inflammation and apoptosis. However, further study is required to determine those possible mechanisms mentioned above and provide direct evidences to confirm the specific target of GA on regulating transporters, inflammation and apoptosis. Nevertheless, our results support the development of GA as a promising drug candidate for treating cholestasis.

## Data Availability

The original contributions presented in the study are included in the article/supplementary material, further inquiries can be directed to the corresponding author.
